# Resveratrol reverses the Warburg effect by targeting the pyruvate dehydrogenase complex in colon cancer cells

**DOI:** 10.1038/s41598-017-07006-0

**Published:** 2017-07-31

**Authors:** Elise Saunier, Samantha Antonio, Anne Regazzetti, Nicolas Auzeil, Olivier Laprévote, Jerry W. Shay, Xavier Coumoul, Robert Barouki, Chantal Benelli, Laurence Huc, Sylvie Bortoli

**Affiliations:** 1 Université Paris Descartes, Sorbonne Paris Cité, UFR des Sciences Fondamentales et Biomédicales, INSERM UMR 1124, F-75006 Paris, France; 2 INSERM UMR 1124, F-75006 Paris, France; 3 Université Paris Descartes, Sorbonne Paris Cité, Faculté des Sciences Pharmaceutiques et Biologiques, Laboratoire C-TAC, Paris, F-75006 France; 4 University of Texas Southwestern Medical Center, Dallas, Texas USA; 5 INRA; TOXALIM (Research Centre in Food Toxicology); 180 Chemin de Tournefeuille, F-31027 Toulouse, France; 6 Université de Toulouse III; INP; ENVT, UPS; TOXALIM, F-31027 Toulouse, France

## Abstract

Resveratrol (RES), a polyphenol found in natural foods, displays anti-oxidant, anti-inflammatory and anti-proliferative properties potentially beneficial in cancers, in particular in the prevention of tumor growth. However, the rapid metabolism of resveratrol strongly limits its bioavailability. The molecular mechanisms sustaining the potential biological activity of low doses of resveratrol has not been extensively studied and, thus, needs better characterization. Here, we show that resveratrol (10 µM, 48 hr) induces both a cell growth arrest and a metabolic reprogramming in colon cancer cells. Resveratrol modifies the lipidomic profile, increases oxidative capacities and decreases glycolysis, in association with a decreased pentose phosphate activity and an increased ATP production. Resveratrol targets the pyruvate dehydrogenase (PDH) complex, a key mitochondrial gatekeeper of energy metabolism, leading to an enhanced PDH activity. Calcium chelation, as well as the blockade of the mitochondrial calcium uniport, prevents the resveratrol-induced augmentation in oxidative capacities and the increased PDH activity suggesting that calcium might play a role in the metabolic shift. We further demonstrate that the inhibition of the CamKKB or the downstream AMPK pathway partly abolished the resveratrol-induced increase of glucose oxidation. This suggests that resveratrol might improve the oxidative capacities of cancer cells through the CamKKB/AMPK pathway.

## Introduction

Cancer cells have energetic needs that differ from those of the tissues from which they are derived and, thus, they modify their use of metabolites to meet these requirements. In fact, most cancer cells exhibit an altered metabolism that is characterized by increased glycolysis and lactate production regardless of the availability of oxygen. This phenomenon is known as the Warburg effect^1^ and it constitutes a hallmark of cancer metabolism. This metabolic switch from oxidative phosphorylation (OXPHOS) to aerobic glycolysis allows cancer cells to produce sufficient energy to survive with limited resources and to divert metabolic intermediates from energy production to the biosynthetic pathways supporting cell proliferation. During the past decade, numerous studies have shown that the metabolic reprogramming of cancer cells is complex and highly flexible. It affects glucose metabolism, together with amino acid and lipid metabolism^2^. Since the glycolytic metabolism of cancer cells is reversible, it could represent a therapeutic target. Thus, the use of agents that mimic energy restriction to selectively target cancer cells which are “addicted” to glycolysis could be a promising therapeutic approach.

Resveratrol is a natural polyphenol which is found mainly in grapes and red wine and is reputed to have beneficial effects for cardiovascular health, obesity, diabetes and cancer. Resveratrol has been shown to modify tumor initiation, promotion and progression^3^ and in a variety of cancer cell lines arrests growth^4–6^.

The mechanism of the antiproliferative effects of resveratrol has been proposed to involve mimicking the effects of caloric restriction. The antitumoral activities of resveratrol could potentially occur through a reduction of glucose uptake and a decrease in the production of lactate^4–8^. Resveratrol, however, can target multiple metabolic enzymes and signaling pathways. Thus, the PI3K signaling pathway has also been reported to be involved in the resveratrol-induced inhibition of glycolysis associated with cell growth arrest in B cell lymphoma^6^, and in breast and colon cancer cells^5, 8^. In these reports, resveratrol was found to negatively regulate some of the proteins and enzymes involved in glucose metabolism such as the glucose transporter GLUT1^8^, phosphofructokinase (PFK1)^4, 6^, hexokinase 2 (HK2), phosphoglycerate mutase (PGAM)^6^, glucose 6 phosphate dehydrogenase (G6PD), transketolase (TKT)^9^ and (PKM2)^7^.

Several lines of evidence suggest that the metabolic effects of resveratrol involve the fuel-sensing AMP-activated kinase (AMPK), a nutrient and energy sensor that maintains energy homeostasis. AMPK is activated by metabolic stresses that decrease ATP levels (by inhibiting its production or accelerating its consumption) that lead to an increase in the amount of AMP, an allosteric activator of AMPK. The activation of AMPK occurs via the phosphorylation of T172 of the α subunit, either by the tumor suppressor Liver Kinase B1 (LKB1) or by the Ca^2+^ Calmodulin kinase kinase B (CamKKB) mediated by an increase in intracellular Ca^2+^ levels^10^. Numerous drugs and xenobiotics, including resveratrol, indirectly activate AMPK by inhibiting ATP synthesis leading to an increase in the level of cellular AMP^11^. Since resveratrol is a potent modulator of many cellular Ca^2+^ signaling pathways^12^, it also might modulate AMPK activity via changes in intracellular Ca^2+^ levels.

Taken together, these findings indicate that resveratrol acts through diverse signaling pathways. Further, they emphasize that enzymes which are involved in the control of the fate of glucose and its metabolites are relevant targets of the polyphenolic compound.

Cancer cells may display differential sensitivity depending on the type of cancer cells^13, 14^. In most of the *in vitro* studies that reported positive effects on cancer cell metabolism^4–8^, doses up to 25 µM resveratrol were employed which could lead to major effects such as oxidative damage and apoptosis^15, 16^. In addition, studies using animal models and humans have shown that the bioavailability of resveratrol does not exceed a few micromolars^17^. The differential sensitivity of cancer cells depending on cell type and resveratrol dose makes it difficult to reconcile the anti-cancer effects of this compound determined from *in vitro* studies with *in vivo* measurement of resveratrol bioavailability. Therefore, we reasoned it is important for *in vitro* studies to better characterize the molecular mechanisms underlying the anti-cancer effects of resveratrol using low rather than saturating doses. In the present study, we decided to expose Caco2 cells to a dose of 10 µM which has been described to induce cell growth arrest in colon cancer cells^18^ without major effects on cell death^19^. We show that resveratrol exposure, at a dose that is compatible with the plasma level of free and metabolized resveratrol reported in rodents and humans after oral consumption of this compound (review in ref. 20), reverses the Warburg metabolic phenotype in colon cancer cells. We suggest that the pyruvate dehydrogenase (PDH) complex, a key enzyme that controls the fate of pyruvate, is a new metabolic target for resveratrol. We also describe the underlying mechanisms that are involved the AMPK signaling pathway and the participation of Ca^2+^ as a second messenger. Finally, we highlight changes in the lipidomic profile of colon cancer cells upon resveratrol exposure.

## Results

### Resveratrol inhibits cell growth of colon cancer cells

**Figure 1:**
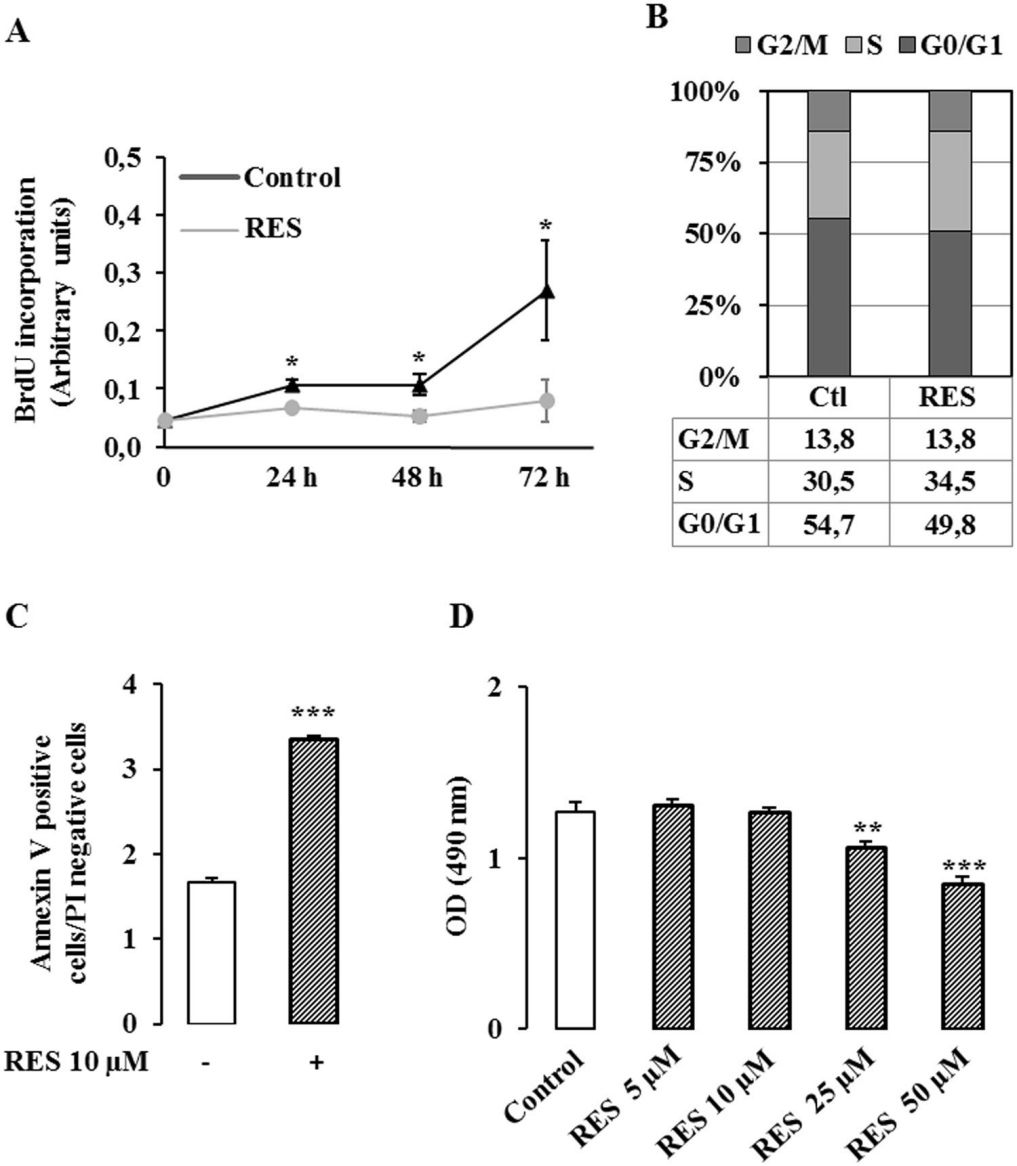
Resveratrol inhibits proliferation without modifying cell viability. (**A**) Caco2 cells were seeded into 96 well plates at 7 500 cells/cm² in DMEM containing 25 mM glucose and supplemented with 5% charcoal-treated serum. Twenty four hours after the plating, cells were treated with 10 µM resveratrol for 24, 48 and 72 hr. Cell proliferation was measured with BrdU according to the manufacturer’s recommendation. (**B**) Caco2 cells were treated with 10 µM resveratrol (RES) for 48 hr and stained with propidium iodide (PI). Quantitative analyses of the percentage of the cells in different phases of the cell cycle were performed by FACS analysis. (**C**) Caco2 cells were treated with 10 µM resveratrol for 48 hr and quantitative analysis of the percentage of the cells positive for annexin V and PI staining were performed by FACS analysis. (**D**) Caco2 cells were treated with 10 µM resveratrol for 48 hr and cell viability was evaluated by MTS assay. The means ± SEM of at least three independent experiments are shown (*p < 0.05, **p < 0.01 vs control, ***p < 0.001 vs control).

The antiproliferative and pro-apoptotic effects of resveratrol have been studied extensively in various several malignant cell lines^9, 21, 22^. The extent of the effects depends upon the doses and the length of treatment with resveratrol and it may differ depending upon the model used. In order to evaluate the toxicity of a relatively low dose of resveratrol, Caco2 colon cancer cells were treated with 10 µM of resveratrol for 24, 48 and 72 hr. We found that resveratrol slowed the proliferation of Caco2 colon cancer cells, as measured by BrdU incorporation, in a time-dependent manner (Fig. 1A). There was a moderate effect at 24 and 48 hr (2-fold change) and a stronger inhibitory effect at 72 hr (3-fold inhibition). As assessed by flow cytometry, exposure to 10 µM resveratrol increased the relative proportion of cells in the S phase of the cell cycle from 30.5% to 34.5% (Fig. 1B) indicating a modest cell cycle effect in S phase. Since resveratrol has been shown to exhibit pro-apoptotic effects, we evaluated the extent of apoptosis by flow cytometric analysis with Annexin V–PI staining. Exposure of Caco2 colon cancer cells to 10 µM resveratrol for 48 hr resulted in a small increase in the apoptotic population, from 1.5 to 3% (Fig. 1C). Finally cell viability was evaluated by MTS assay. Although a treatment of cells with 10 µM resveratrol for 48 hr had no effect on viability, higher doses of resveratrol decreased cell viability (Fig. 1D). Taken together, these observations show that treatment of cells with 10 µM resveratrol for 48 hr slowed proliferation but did not induce massive cell death.

### Resveratrol elicits a metabolic shift from glycolysis to OXPHOS in cancer cells

**Figure 2:**
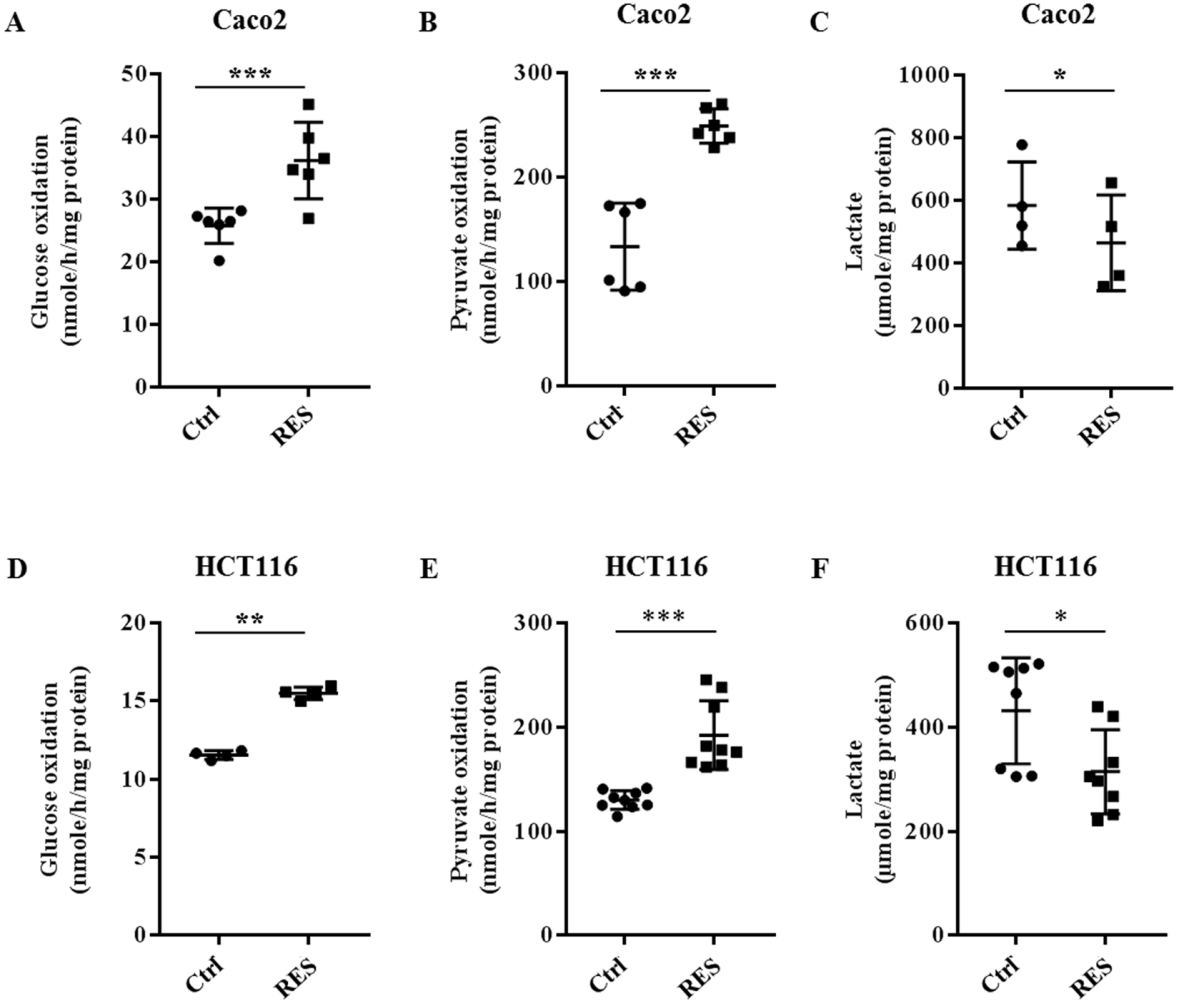
Resveratrol induces a shift toward oxidative metabolism in colon cancer cells. After treatment with 10 µM resveratrol (RES) for 48 hr, oxidative capacity was evaluated as following: subconfluent cancer cells were isolated and 10^6^ cells were incubated in Krebs-Ringer phosphate buffer supplemented with 5 mM [U^14^C]-glucose (isotopic dilution 1/1000) (**A**,**D**) or with 5 mM ^14^C_1_-pyruvate (isotopic dilution 1/250) (**B**,**E**). ^14^CO_2_ was recovered as described in the Material and Methods section. The lactate secretion in the medium was measured under the same conditions (**C**,**F**) as described in the Material and Methods section. (*p < 0,05, **p < 0,01, ***p < 0.001 vs control).

Since cell growth is directly dependent on cell metabolism, we analyzed the effects of different doses of resveratrol (1 to 50 µM, 48 hr) on glucose utilization in colon cancer cells. We showed that only an exposure to 10 µM resveratrol led to a significant increase (65%) of the ability of Caco2 cancer cells to oxidize glucose (Supplementary Figure S1 and Fig. 2A). resveratrol similarly affected glucose oxidation in HCT116 human colon cancer cells (+41%) (Fig. 2D) and MCF7 human breast cancer cells (+51%) (Supplementary Figure S2A). These observations demonstrate that the effect of resveratrol on cancer cell metabolism is not restricted to colon cancer cells but affects the metabolism of other types of cancer cells. Resveratrol also increased the oxidation of pyruvate (51% in Caco2 cells, 48% in HCT116 cells) as shown in Fig. 2B,E. Consistent with the increased oxidative capacity of resveratrol-treated cells, we observed that resveratrol (10 µM, 48 hr) decreased the production of lactate, an end product of non-oxidative glucose metabolism in colon cancer cells (Fig. 2C,F) and in breast cancer cells (Supplementary Figure S2B).

**Figure 3:**
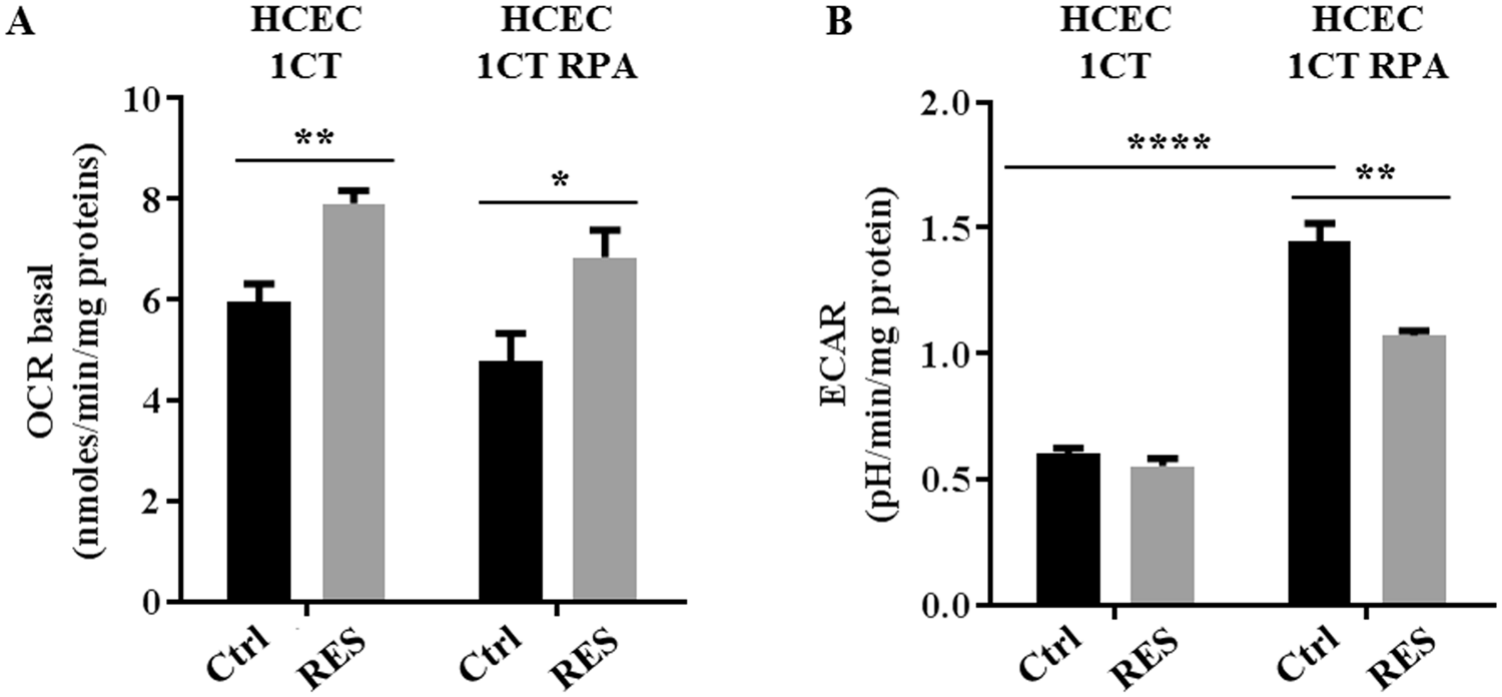
Resveratrol promotes a shift from respiration to glycolysis in cancer-like cells, but not in normal colonocytes. HCEC 1CT and HCEC 1CT RPA cells were treated with 10 µM resveratrol (RES) for 48 hr and then OCR (**A**) and ECAR (**B**) were estimated using Seahorse technology, as described in the Material and Methods section. The means ± SEM are shown for at three independent experiments performed in quadruplicate (*p < 0.05, **p < 0.01, ****p < 0.0001 vs control).

In order to determine whether the effect of resveratrol on metabolism is specific to cancer cells or would also affect normal cells, we used a human normal colonic epithelial cell line HCEC 1CT derived from a healthy patient that was been immortalized with cyclin-dependent kinase 4 (cdk4) and the catalytic component of the human ribonucleoprotein enzyme telomerase (hTERT). These cells are normal diploid and do not have any tumorigenic properties^23^. The metabolic effect resveratrol has been compared between normal HCEC 1CT and the isogenic derivative cell line HCEC 1CT RPA, a more tumor progressed cell line that is characterized by 90% knockdown of APC and TP53, and the ectopic expression of KRAS, mimicking the three major alterations found in colorectal cancers^23–26^. The basal level of respiration was 6.0 nmol/h/mg protein in normal colonic epithelial HCEC 1CT cells, and 4.8 nmol/h/mg protein in HCEC 1CT RPA cells. Resveratrol exposure (10 µM, 48 hr) induced a significant increase of oxygen consumption rate (OCR) both in normal colonic epithelial HCEC 1CT cells (32.7%) and in cancer progressed HCEC 1CT RPA cells (43.2%) (Fig. 3A). As shown in Fig. 3B, the basal level of extracellular acidification rate (ECAR) was significantly higher in cancer progressed HCEC 1CT RPA cells (+241%) compared to the normal HCEC 1CT cells, suggesting a more glycolytic phenotype. Following treatment with resveratrol (10 µM, 48 hr), the ECAR was unchanged in normal HCEC 1CT cells, whereas it was significantly reduced (31%) in HCEC 1CT RPA cells (Fig. 3B), supporting the hypothesis of a shift from glycolysis to OXPHOS in colon cancer cells, but not in normal colonic epithelial cells.

**Figure 4:**
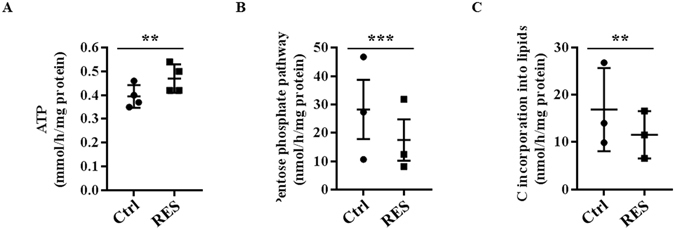
Resveratrol modifies glucose utilization in colon cancer cells. (**A**) Caco2 cells were treated with 10 µM resveratrol (RES) for 48 hr and ATP production was estimated by using ATP Bioluminescence assay kit II. (**B**) After a 48 hour-treatment with 10 µM, subconfluent Caco2 cells were isolated and 10^6^ cells were incubated in Krebs-Ringer phosphate buffer supplemented with 5 mM [^14^C_1_]-glucose (isotopic dilution 1/1000) or [^14^C_6_]-glucose (isotopic dilution 1/100). ^14^CO_2_ was recovered and pentose phosphate pathway (PPP) activity was calculated as described in the Material and Methods section. (**C**) After treatment with resveratrol, cells were isolated and incubated in Krebs-Ringer phosphate buffer supplemented with 5 mM [U^14^C]-glucose (isotopic dilution 1/1000). The incorporation of carbons from glucose was measured in total lipids according to Bligh and Dyer^57^ as described in the Material and Methods section. The means ± SEM are shown for at least three independent experiments performed in triplicate (**p < 0.01, ***p < 0.001 vs control).

Resveratrol-induced changes in glucose metabolism were associated with a significant increase (20%) in ATP production (Fig. 4A). To further characterize the glucose metabolism in resveratrol-treated cells, we investigated the activity of the pentose phosphate pathway (PPP). This pathway represents a major advantage for enhanced glycolysis for tumor cells by providing NADPH for fatty acid synthesis and for the cell’s antioxidant defenses against a hostile microenvironment. We found that resveratrol decreases the pentose phosphate pathway activity by 36% (Fig. 4B) and reduces significantly (35%) the utilization of glucose to make lipids (Fig. 4C). Taken together, these observations indicate that exposure to resveratrol leads to a metabolic reorientation from aerobic glycolysis toward OXPHOS.

### Resveratrol alters the lipidomic profile of colon cancer cells

**Figure 5:**
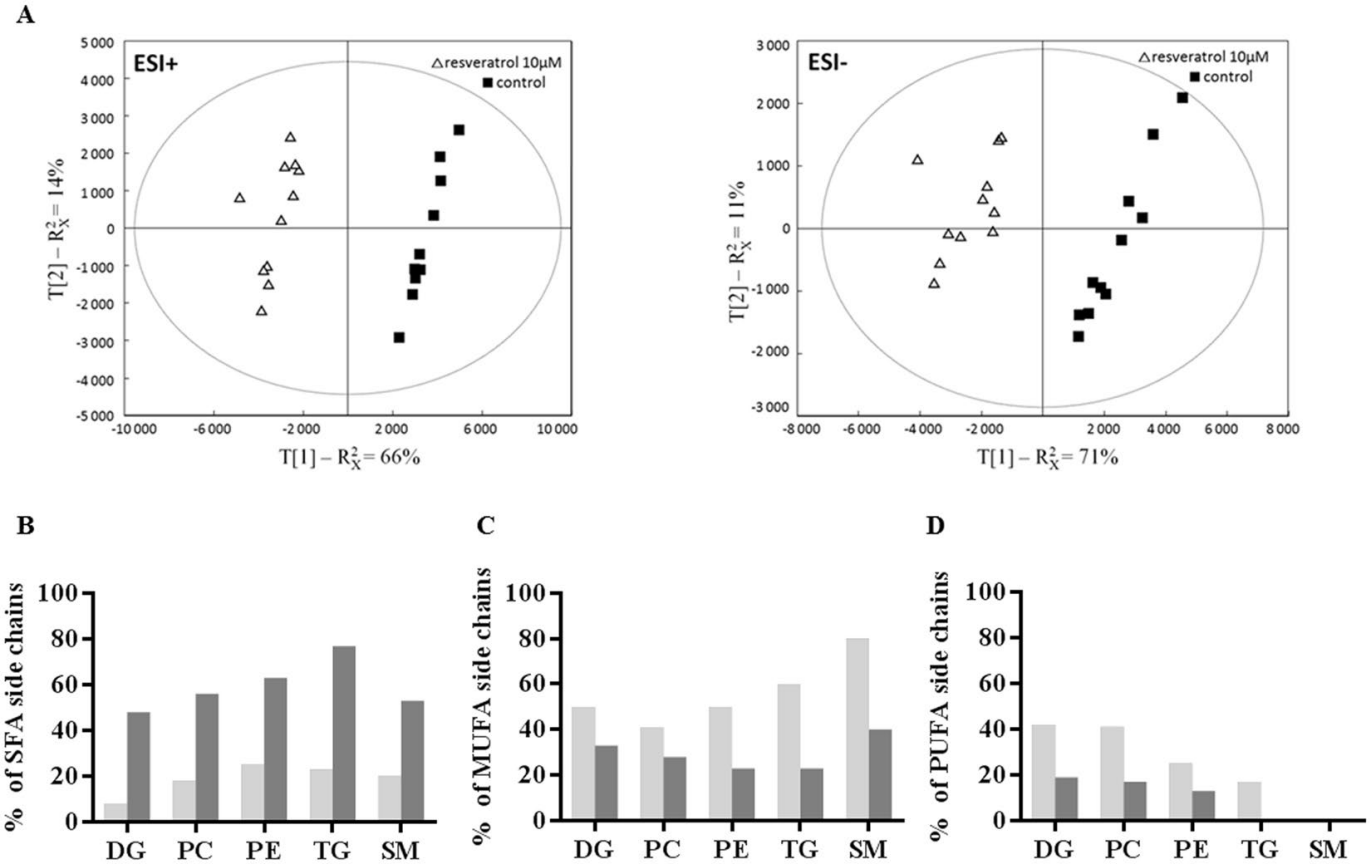
Resveratrol modulates the lipidomic profile of colon cancer cells. Caco2 cells from 12 different passages were treated with 10 µM resveratrol (RES) for 48 hr. After lipid extraction according to Bligh and Dyer^57^, lipid extracts were analysis as described in Materials and Methods. (**A**) PCA score plots of resveratrol treated- (Δ, n = 12) and control cells (■, n = 12) were built from first and second component of PCA models generate from data acquired in UPLC-MS in ESI + (left) and ESI- (right) ion modes. Distribution of lipid species with (**B**) saturated (SFA), (**C**) monounsaturated (MUFA) and (**D**) polyunsaturated (PUFA) fatty acid side chains, in lipids showing an increased (dark grey bars) or a decreased (light grey bars) relative abundance following an exposure to resveratrol (10 µM, 48 hr).

Although disregarded in the past, alterations of lipid metabolism that occur during tumor development are now becoming well recognized. In order to characterize the effects of RES on lipid metabolic profiles, we performed lipidomic analysis of colon cancer cells following a resveratrol exposure (10 µM, 48 hr). The score plots of PCA models indicate a clear separation between control and resveratrol-treated cells (10 µM, 48 hr) (Fig. 5A). This separation was confirmed on the PLS-DA and OPLS-DA score plots. These results indicate that resveratrol exposure leads to substantial modifications of the lipid composition in Caco2 colon cancer cells (Supplementary Table 1).

In lipid families such as diacylglycerol (DG), triacylglycerol (TG), phosphatidylcholine (PC), phosphatidylinositol (PI) and sphingomyelin (SM), certain species showed an increase in their relative abundance while others were decreased in Caco2 cells. For example, in the PC family, 8 lipid species were decreased and 7 were increased (Supplementary Table 1).

The lipid species exhibiting a decreased abundance in resveratrol-treated cells, contained more unsaturated fatty acid (FA) and less saturated FA side chains when compared to the lipid species exhibiting an increased abundance (Fig. 5B, 5C, 5D). Conversely, the lipids whose level was upregulated by resveratrol contained more saturated FA and less unsaturated FA side chains (Fig. 5B, 5C, 5D). The modified balance between saturated and unsaturated fatty acids that composed the side chains indicates that resveratrol might induce a decrease in the global content of unsaturated fatty acids in lipids.

Resveratrol also induced changes in phosphatidylethanolamine (PE) and ceramides (Cer) families, with an upregulation of almost all the species. Indeed, the relative abundance of 18 PE and 17 Cer was increased whereas the level of 2 PE and 2 Cer was decreased (Supplementary Table 1). Among the above ceramides, Cer(42:1), Cer(42,2), Cer(44:2), GlcCer(40:2), GlcCer(42:1), GlcCer(42,2) and GlcCer(44:2) were increased, in relation with the decrease observed for the corresponding sphingomyelins. The sphingomyelin/ceramide pathway has been implicated in the regulation of several tumorigenic-related processes including cell proliferation and apoptosis. An increase of ceramide levels is consistent with an anti-tumorigenic effect of resveratrol.

### Resveratrol does not modify the expression of key proteins involved in glucose utilization but does enhance PDH activity

We then determined whether the resveratrol-induced metabolic changes occurred through modifications in the level of expression of key proteins involved in glucose metabolism. Resveratrol did not modulate the level of the glucose transporter GLUT1, the rate-limiting enzyme of the pentose phosphate pathway glucose 6-phosphate dehydrogenase (G6PD), the enzyme that catalyzes the last step of the glycolysis pyruvate kinase M2 (PKM2), or the lactate dehydrogenase A (LDHA) which converts pyruvate to lactate (Supplementary Figure S3A). Since the activities of PKM2 and LDHA are regulated by phosphorylation, the level of the phosphorylated forms of these enzymes was also evaluated and no change in phosphorylation of either enzyme was detected (Supplementary Figure S3B).

Pyruvate dehydrogenase (PDH) is a key mitochondrial enzyme that links glycolysis to the TCA cycle. PDH activity is regulated by the reversible phosphorylation of 3 serines of the PDHE1α subunit (S232, S293 and S300) by pyruvate dehydrogenase kinases (PDK1-4) that decreases the use of pyruvate in the TCA cycle. Dephosphorylation of these sites by pyruvate dehydrogenase phosphatases (PDP1-2) stimulates PDH activity^27^. PDK1, the predominant isoform of PDKs in cancer cells, was recently identified as a potential target for anti-glycolytic therapy in cancer^28^.

**Figure 6:**
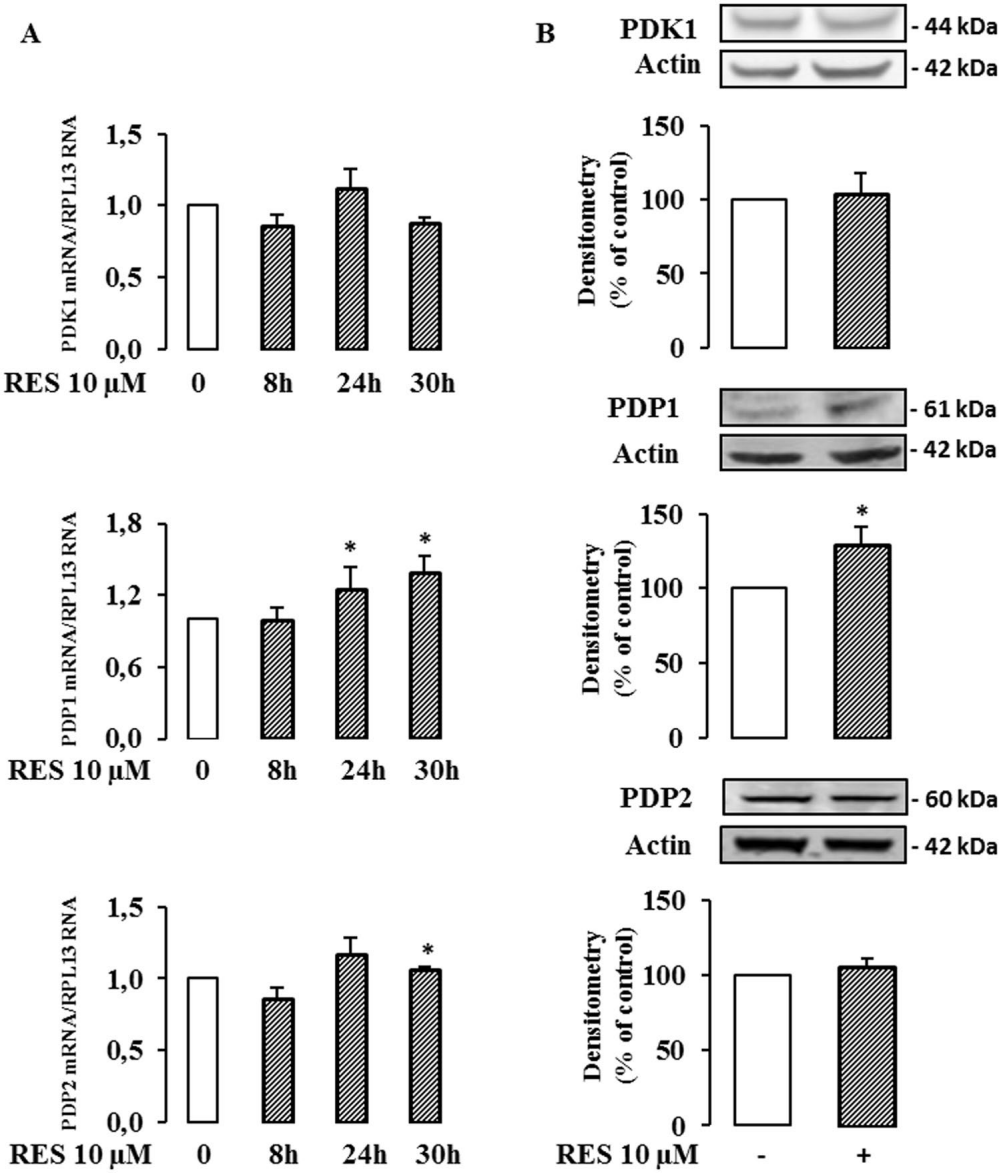
Resveratrol modulates PDP1 level in cancer cells. Caco2 cells were cultured at subconfluence in 5% charcoal-treated serum in DMEM 24 hr before and during the treatment with 10 µM resveratrol (RES) for the indicated times. (**A**) mRNA levels of PDK1, PDP1 and PDP2 were evaluated by real time RT-PCR and normalized using RPL13 RNA. (**B**) After a 48 hr exposure with resveratrol 10 µM, total cell lysates were collected using RIPA buffer supplemented with protease and phosphatase inhibitors and analyzed by Western blot using antibodies against PDK1, PDP1 and actin for normalization. Data are the means ± SEM of at least 3 independent experiments (*p < 0.05).

**Figure 7:**
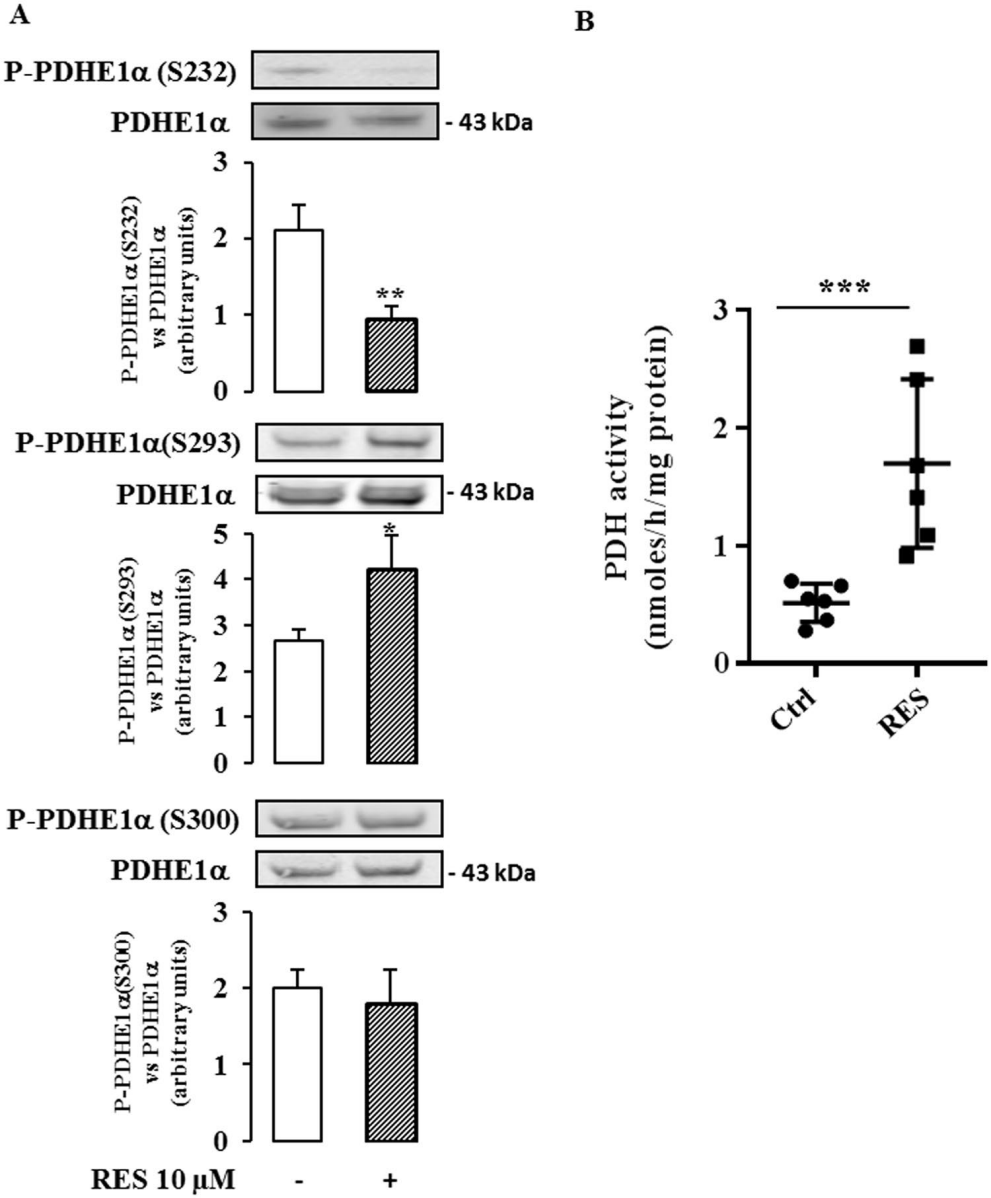
Resveratrol modulates PDH actvity in cancer cells. Caco2 cells were cultured at subconfluence in 5% charcoal-treated serum in DMEM 24 hr before and during the treatment with 10 µM resveratrol (RES) for 48 hr. (**A**) Total cell lysates were collected using RIPA buffer supplemented with protease and phosphatase inhibitors and analyzed by Western blot using antibodies against P-PDHE1α(S232), P-PDHE1α(S293) and P-PDHE1α(S300), and PDHE1α and actin for normalization. Data are the means ± SEM of at least 3 independent experiments (*p < 0.05, **p < 0.01). (**B**) Caco2 cells were cultured at subconfluence in 5% charcoal-treated serum in DMEM 24 hr before and during the treatment with 10 µM resveratrol for 48 hr. PDH activity was assayed by the release of ^14^CO_2_ from [^14^C_1_]-pyruvate according to Geoffroy *et al*.^58^, as described in the Material and Methods section. Data are the means ± SEM of 6 independent experiments (***p < 0.001).

We found that the expression of the gene encoding PDK1 (Fig. 6A upper panel) and the corresponding protein (Fig. 6B upper panel) were not modified by a treatment with 10 µM resveratrol for 48 hr. In contrast, resveratrol exposure significantly enhanced the expression of PDP1 mRNA (25% at 24 hr, 38% at 30 hr, Fig. 6A middle panel), as well as the expression of the PDP1 protein (29% at 48 hr, Fig. 6B middle panel). The expression of PDP2 mRNA was only significantly altered after a 24 hour-exposure to resveratrol (Fig. 6A bottom panel), but the protein level was unchanged (Fig. 6B bottom panel). We, therefore, investigated the effect of resveratrol on the level of phosphorylation of the PDHE1α subunit, using specific antibodies for the 3 phosphorylated serine residues. There was a significant decrease in the phosphorylation of S232, a slight increase in the phosphorylation of S293 and no significant change in phosphorylation of S300 (Fig. 7A). Finally, we measured the activity of the PDH complex in colon cancer cells following treatment with 10 µM resveratrol for 48 hr using [^14^C_1_]-pyruvate and we observed a marked increase (x 2.6) in PDH complex activity (Fig. 7B).

Altogether, these data can be interpreted to suggest that the PDH complex participates in the resveratrol-induced reorientation of the glycolytic flux at least by regulating PDP1 expression.

### Metabolic effects of resveratrol on glucose metabolism are associated with a modulation of Ca^2+^ flux

**Figure 8:**
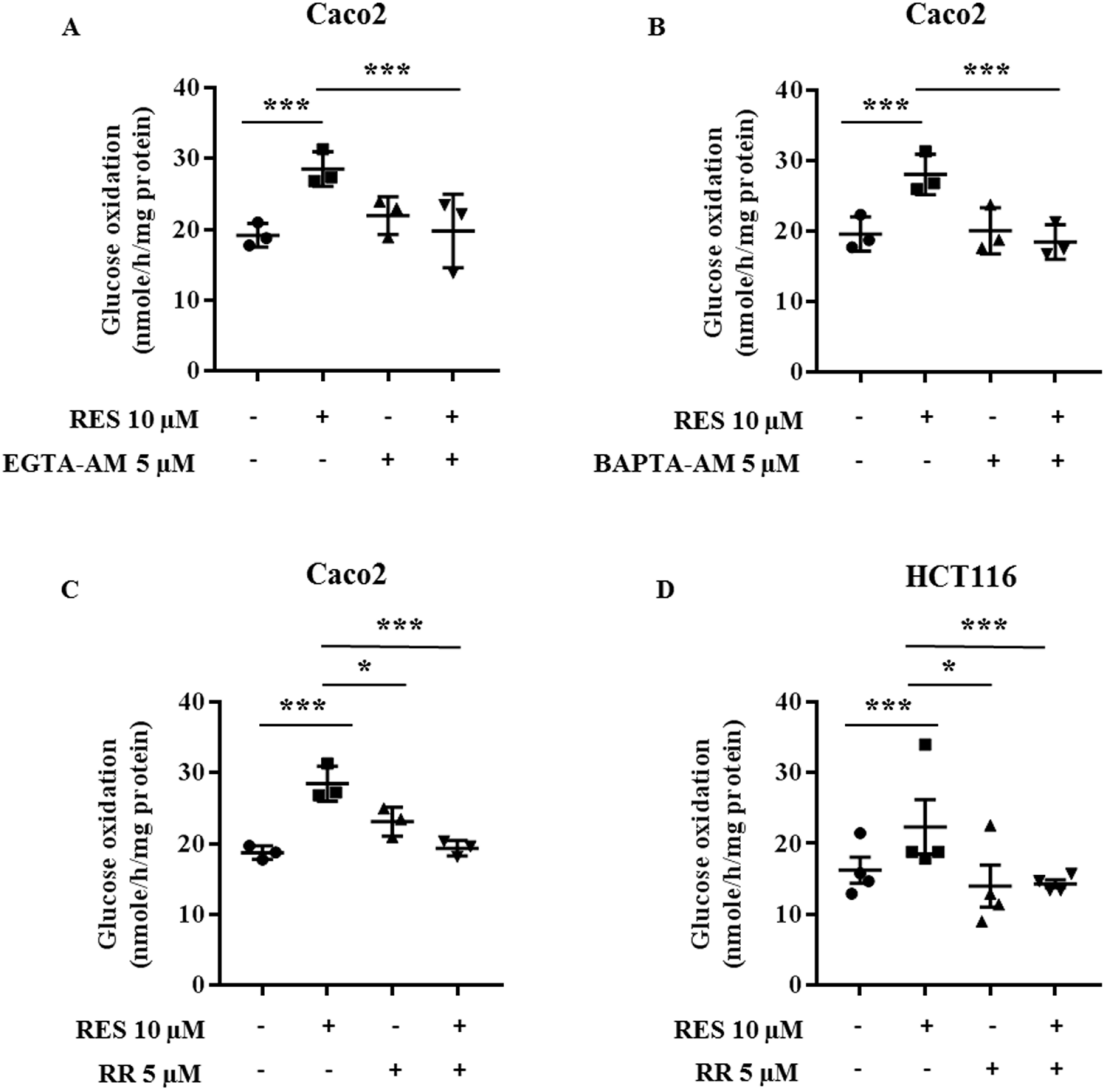
Metabolic effects of resveratrol on glucose metabolism are related to a modulation of calcium flux in colon cancer cells. Subconfluent colon cancer cells were treated with 5 µM EGTA-AM (**A**, n = 3), 5 µM BAPTA-AM (**B**, n = 3) or 5 µM Ruthenium Red (**C**, n = 3; **D**, n = 4), 1 hour before and during a 48 hour-treatment with 10 µM resveratrol (RES). Cells were isolated and 10^6^ cells were incubated in Krebs-Ringer phosphate buffer supplemented with 5 mM [U^14^C]-glucose (isotopic dilution 1/1000). ^14^CO_2_ was recovered as described in the Material and Methods section.

**Figure 9:**
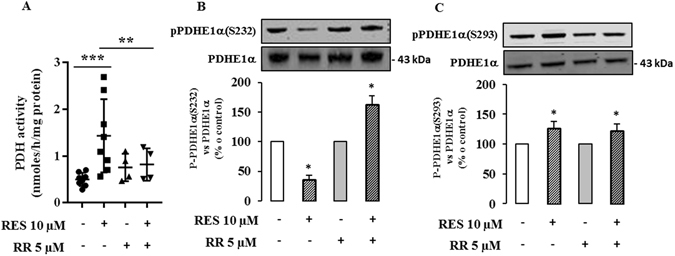
Calcium modulates resveratrol-induced increase of PDH activity. (**A**) Caco2 cells were treated 5 µM RR and then with 10 µM resveratrol (RES) for 48 hr. PDH activity was measured as in the Material and Methods section (n = 4 to 8). Total cell lysates were collected from cells using RIPA buffer supplemented with protease and phosphatase inhibitors. Lysates were analyzed by Western blot using antibodies against (**B**) P-PDHE1α (S232) (n = 3) and (**C**) P-PDHE1α (S293) (n = 6). (*p < 0.05, **p < 0.01, ***p < 0.001 vs control).

Resveratrol is a potent regulator of intracellular ion homeostasis, including modulation of the intracellular Ca^2+^ concentration. Ca^2+^ is a second messenger known to be a positive effector of mitochondrial function. Thus, we investigated the role of Ca^2+^ on the effect of resveratrol on glucose metabolism. Chelation of Ca^2+^ either by EDTA-AM (Fig. 8A) or by BAPTA-AM (Fig. 8B) totally abolished the increase in glucose oxidation after resveratrol treatment of colon cancer cells. This suggests that Ca^2+^ is a key mediator of the effect of resveratrol on the oxidative capacity of colon cancer cells. Moreover, pre-treatment for 1 hr with ruthenium red (RR), an inhibitor of a mitochondrial Ca^2+^ uniport, before a 48 hour-treatment with resveratrol, prevented the resveratrol-induced increase of glucose oxidation (Fig. 8C,D). In addition, the blockade of Ca^2+^ transport into the mitochondria by RR prevented the increase of PDH activity upon resveratrol exposure (Fig. 9A). This may occur, at least in part, through a reversion of the resveratrol-induced decrease in P-PDHE1α(S232) (Fig. 9B) whereas RR failed to cancel the enhancement of P-PDHE1α(S293) upon resveratrol exposure (Fig. 9C). We interpret these observations to suggest that Ca^2+^ plays a role in the resveratrol-mediated increase of the oxidative capacities of cancer cells via the PDH complex. It also suggests that Ca^2+^ might be involved in the phosphorylation of S232 but not in the phosphorylation of S293.

### The increase of glucose oxidation in response to resveratrol involves the AMPK signaling pathway in colon cancer cells

**Figure 10:**
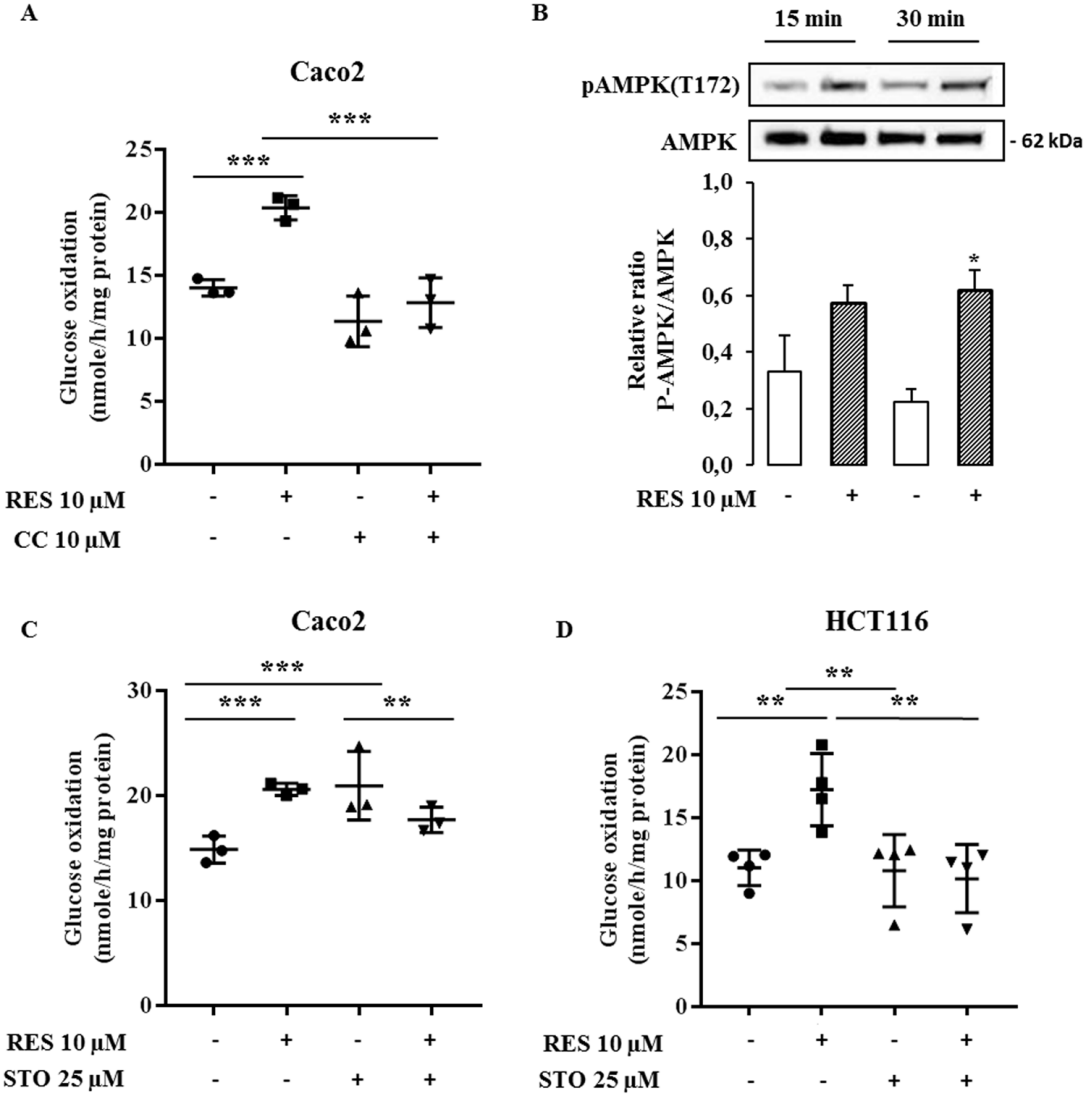
AMPK signaling is involved in resveratrol-induced metabolic shift in colon cancer cells. Subconfluent colon cancer cells were treated with 10 µM of compound C (CC), an AMPK inhibitor (**A**) or 25 µM of STO-609 (STO), a CAMKK inhibitor (**C**,**D**), 1 hour before and during a 48 hour-treatment with 10 µM resveratrol (RES). Cells were isolated and 10^6^ cells were incubated in Krebs-Ringer phosphate buffer supplemented with 5 mM [U^14^C]-glucose (isotopic dilution 1/1000). ^14^CO_2_ was recovered as described in the Material and Methods section. (**B**) Total cell lysates were collected from Caco2 cells treated with 10 µM resveratrol for 30 minutes and 1 hour, using RIPA buffer supplemented with protease and phosphatase inhibitors. Lysates were analyzed by Western blot using antibodies against P-AMPK(T172) and AMPK. Results are the means ± SEM of at least 3 independent experiments each performed in triplicate (*p < 0.05, **p < 0.01, ***p < 0.001 vs control).

To further elucidate the regulatory pathways implicated in the effect of resveratrol on cancer cell metabolism, we examined the AMPK signaling pathway. AMPK is an important regulator of energy homeostasis and it is involved in glucose and lipid metabolism. Furthermore, CamKKB can activate AMPK by phosphorylation following changes in the intracellular Ca^2+^ level. It also has been suggested that activated AMPK could participate in the inhibition of cell proliferation and tumor progression (for a review see ref. 29). In order to investigate the involvement of the AMPK signaling pathway in the metabolic effects of resveratrol, we used the compound C, an inhibitor of AMPK, one hour before a 48 hour-treatment of the cells with 10 µM resveratrol. We found that the blockade of AMPK activation prevented the resveratrol-induced improvement in the oxidative capacities of cancer cells (Fig. 10A). AMPK activation involves phosphorylation of the T172 of the AMPKα2 subunit. We observed that resveratrol treatment induced a rapid increase of the T172 phosphorylation (Fig. 10B) which suggests that the activation of AMPK is required for the resveratrol-dependent metabolic shift. When the cells were treated with STO-609, an inhibitor of CamKKB, one hour before a 48 hour-treatment of cells with resveratrol, resveratrol no longer had a positive effect on the oxidative capacities of the colon cancer cells (Fig. 10C,D). Taking together, these data can be interpreted to suggest that resveratrol promotes the oxidative capacities of colon cancer cells through a CamKKB signaling pathway.

## Discussion

In the present study, we show that resveratrol, at a concentration as low as 10 µM, reduces colon cancer cell proliferation and induces a metabolic reprograming leading to an increase of glucose oxidation, a decrease of lactate production, a decrease of pentose phosphate activity and a decrease of lipid synthesis from glucose. Under the conditions used in the study, no significant decrease in cell viability or increase of apoptosis was detected.

In several *in vitro* studies and in animal models, resveratrol has been demonstrated to be an antitumor and chemopreventive agent. Further, it affects cellular proliferation through its action on tumor initiation, promotion, and progression^3, 30^. These properties have been explained mainly by its activities in cell cycle control and the induction of apoptosis^31, 32^. In numerous *in vitro* studies, resveratrol, at doses from 10 µM to up to 200 µM, has been shown to affect cell growth and/or viability of a variety of tumor cells^13–16^. In our study, we have shown that resveratrol had a significant effect on cell viability of Caco2 colon cancer cells from 25 µM that is consistent with others reports^33, 34^. Actually, the sensitivity of cancer cells from various origin (breast, prostate, blood, brain, colon) upon resveratrol exposure *in vitro* depends on the doses and the types of cancer cells^13, 16, 35^. Furthermore, differential responsiveness of cancer cell lines to resveratrol could also occur for a given cancer cell type. For example, resveratrol significantly decreased cell growth from 40 µM in T24 human bladder cancer, and from 100 µM in EJ human bladder cancer^14^. Here we observed that a dose of 10 µM resveratrol can limit Caco2 cancer cell proliferation without major effects on cell death. Similar results have been reported in other colon cancer cell lines (namely HCT116, HT29 and SW480 cells). Resveratrol inhibited cell cycle progression by enhancing the levels of cyclin E and cyclin A^21, 22^ and by decreasing cyclin D1^9, 21^ which suggests that resveratrol, at doses ≤50 µM, exerts mainly cytostatic but not cytotoxic effects on colon cancer cells.

Confirmation of beneficial effects in humans through randomized clinical trials has been limited and the results are moderate, probably due to the low bioavailibity of resveratrol in humans. In rodents and in humans, following oral consumption, resveratrol (70–80%) is quickly absorbed via passive diffusion in the intestine^36–38^, and it is rapidly metabolized to the conjugation products resveratrol-3-O-glucuronide and resveratrol-3-O-sulfate^38, 39^. A good safety has been reported in healthy volunteers after receiving for 29 days daily doses of resveratrol 0,5 to 5 g/day. This study showed that repeated administration of high dose of resveratrol generate micromolar concentrations of resveratrol and much higher plasmatic levels of conjugates^40^. In a more recent study, pharmacokinetic properties of resveratrol have been studied by the administration of an isotopic dilution of ^14^C-resveratrol (1 g/1 single intake) to human healthy volunteers. The average maximal plasma concentration reached 137 µM after 1 hr and 14 µM after 24 hr^17^. Furthermore, they showed that resveratrol species reached the intestinal tissue of all patients^17^. These results are consistent with the fact that resveratrol and its metabolites are mainly eliminated through feces^41^, that could explain their presence at relatively high concentration in colorectal tissue^42^. High level of resveratrol has also been observed in the intestine of mice after oral administration^43^ and resveratrol is highly absorbed and metabolized in human Caco2 cells^36^, suggesting colon cancer cells as a major target for anti-cancer properties. In our study, we showed that a pharmacologically achievable dose of resveratrol induces a metabolic reprogramming in both normal (HCEC 1CT cells) and tumorigenic cells (HCEC 1CT RPA, Caco2, HCT116). In cancer cells, resveratrol promotes a shift from a glycolytic phenotype toward a more oxidative metabolism. As an enhanced glycolysis is frequently related to the aggressiveness and invasive properties of cancer cells^44^, our new data also support the hypothesis of an anti-tumoral action of resveratrol *via* a metabolic reversion. The effects of resveratrol are complex, some are specific to tumorigenic cells (decreased glycolysis), while others are not (increased oxidative capacities).

Metabolic reprogramming is a central feature of cancer cells. In addition to alterations in glucose and amino acid metabolism, cancer cells frequently display changes in lipid abundance and composition. In particular, it is well known that overexpression of lipogenic genes and overall lipogenesis are hallmarks of cancer cells (for a review see ref. 45) that predominantly esterified fatty acids to phospholipids for membrane lipid synthesis, which promotes cell replication, rather than used for triglyceride energy storage^45^. In our study, we highlighted that resveratrol decreased the activity of the pentose phosphate pathway together with the utilization of glucose to produce lipids. The concomitance of these events appears to be relevant since the pentose phosphate pathway provides NADPH, a cofactor that plays a major role in lipid synthesis as a substrate of the fatty acid synthase (FAS). Our observations are in accordance to previous reports showing that resveratrol suppressed tumor growth of breast cancer cells and pancreatic adenocarninoma cells by a downregulation of FAS and a decreased lipogenesis^46, 47^.

Lipid repertory is also modified in cancer cells, leading to alterations of membrane fluidity, signal transduction and chemotherapeutic drug responsiveness. For example, ceramides are key components of complex sphingolipids and regulate various cellular processes including cell proliferation, differentiation and apoptosis^48^. The level of intracellular ceramides is regulated via a *de novo* biosynthesis from serine and palmitoyl-CoA as well as by the sphingomyelinase-mediated hydrolysis of sphingomyelin. In MDA-MB231 breast cancer cells, resveratrol exposure led to both *de novo* ceramide biosynthesis associated with the activation of serine palmitoyltransferase, the key enzyme of *de novo* ceramide biosynthetic pathway, and a sphingomyelin hydrolysis linked to the activation of neutral sphingomyelinase, the main enzyme involved in the sphingomyelin/ceramide pathway^49^. Resveratrol also induced an increase of ceramides and a decrease of sphingomyelins, via a transcriptional upregulation of the acid sphingomyelinase in HCT116 colon cancer cells and in K562 erythroleukemia cells^50^. In Caco2 colon cancer cells, a tight correlation exists between the resveratrol-induced antiproliferative effect and a strong increase of endogenous ceramides, associated with an inhibition of ornithine decarboxylase activity^51^. In the present studies, we showed that resveratrol induced an augmentation of ceramides and a decrease of sphingomyelins. Considering the role of sphingomyelins and ceramides as second messengers in many intracellular events such as cell growth, differentiation and apoptosis, one can assume that resveratrol might profoundly reshape the signaling pathways involved in the fate of cancer cells. We also showed that resveratrol exposure led to an increase of the balance saturated vs unsaturated fatty acids (SFA/UFA) that composed the side chains of a large variety of lipids (including phospholipids and triglycerides). Recently, the silencing of stearyl-coA desaturase 1 (SCD, involved in the formation of UFA from SFA) in prostate orthografts has been linked to a blockade of tumor growth and an increased animal survival^52^, supporting the hypothesis of a role of SCD in the resveratrol-induced changes in the SFA/UFA ratio. This is consistent with previous studies that show that resveratrol exposure induces a decrease of LXRα-mediated hepatic lipogenesis through a downregulation of FAS and SCD^53^, and a decrease of the expression of SCD in the liver of high fat diet mice^54^ and in human hepatocarcinoma HepG2 cells^55^. Further studies are needed to better understand the consequences of these lipid alterations on membrane homeostasis and mitochondrial function, in particular in the context of cancer progression.

The emergence of metabolic enzymes as important regulators of cancer cell growth shows that metabolic control is a key element for tumor progression. It is widely accepted that tumors display enhanced glycolytic activity and impaired OXPHOS (Warburg effect). Several strategies targeting metabolic enzymes have demonstrated that the reorientation of cancer cell metabolism from glycolysis to OXPHOS can lead to diminished cell survival, invasiveness and tumor growth^56–58^. Therefore, glycolysis appears to be a promising domain for specific anti-cancer therapy. In support of its candidacy as a promoter of a metabolic switch, resveratrol has been associated with impaired transport and/or utilization of glucose, at the level of transcription and/or translation of crucial metabolic enzymes, such as pyruvate kinase M2^7^, 6-phosphofructo-1 kinase^6^, hexokinase 2^6^, glucose 6-phosphate dehydrogenase^9^, transketolase^9^ or glucose transporter GLUT1^8^. Under the conditions used in our study, resveratrol does not affect the expression (mRNA or protein levels) of several key enzymes of energy metabolism and suggests that other targets or other levels of regulation might be implicated in the effects of resveratrol on cancer cell metabolism. A candidate is pyruvate dehydrogenase (PDH), the activity of which is increased by resveratrol *in vivo*, in cardiac muscle of diabetic rats^59^ and in skeletal muscle of aged mice^60^. PDH is a mitochondrial complex that acts as a gatekeeper that can regulate pyruvate flux from the cytoplasm to the mitochondria by coupling glycolysis to OXPHOS. The main mechanism for the control of PDH activity occurs by phosphorylation and dephosphorylation of the PDHE1α subunit via specific kinases (PDK1-4) and phosphatases (PDP1-2)^47^. The activation/inactivation of the PDH complex plays a major role in the metabolic adaptations that occur during the acquisition of the tumor metabolic phenotype^27^. Several reports have suggested that the metabolic changes that occur in cancer cells might be due, in part, to the attenuation of mitochondrial function subsequent to inhibition of PDH activity^56, 61–63^. A key regulator of PDH activity is PDK1, which is known to be overexpressed in several cancer cells^28, 56, 61^. The regulation of PDH activity through a modulation of PDPs is less well known. In this study we showed that resveratrol did not modify the amount of PDK1 but did upregulate the Ca^2+^-sensitive PDP1 which might contribute to the enhanced PDH activity upon resveratrol exposure. As resveratrol induced a differential phosphorylation of serine residues of PDHE1α, with a decrease of P-PDHE1α(S232) and an increase of P-PDHE1α(S293) together with an enhancement of PDH activity, this suggests a major role of S232 in PDH activation upon resveratrol exposure.

Among the different regulators of mitochondrial function, mitochondrial Ca^2+^ flux is known to modulate key physiological processes^64^ and, in particular, the activities of several mitochondrial dehydrogenases such as FAD-glycerol phosphate dehydrogenase, NAD^+^-isocitrate dehydrogenase, oxoglutarate dehydrogenase, and phosphatases such as PDP1^65^. It has been proposed that resveratrol controls Ca^2+^ influx through the release of intracellular stores and that it activates downstream Ca^2+^-sensitive molecules^12^. We found that blocking the uniporter of the inner mitochondrial membrane with an inhibitor of Ca^2+^ uptake into mitochondria, completely abolished the effects of resveratrol on glucose oxidation as well as on the activation of PDH. Similar results were obtained when the cells were treated with the Ca^2+^ chelators EGTA-AM and BAPTA-AM. These results strongly suggest that Ca^2+^ signaling is involved in the resveratrol-induced switch from glycolysis to OXPHOS, probably through the regulation of PDH activity. The blockade of Ca^2+^ entry into the mitochondria abolished the activation of PDH upon resveratrol exposure. This effect was associated with a reversion of the resveratrol-induced decrease of P-PDHE1α(S232), whereas the increase of P-PDHE1α(S293) upon resveratrol exposure was unaffected by Ca^2+^ blockade. It is known that Ca^2+^ can markedly increase PDH activity by enhancing PDP activity^66^. Among the two PDP isoforms, PDP1 has been described to be activated by Ca^2+^ ions, as extensively described by Denton^65^. In this studies it was reported in particular, that Ca^2+^ is required for the binding of PDP to the lipoyl group of the E2 subunit of the PDH complex^65^. Our hypothesis is that resveratrol may regulate the activity of the PDH complex through 2 distinct mechanisms: an increased expression of PDP1 and an augmentation of the Ca^2+^ flux from the cytosol to the mitochondria that might induce an enhancement of PDP1 activity.

The detailed mechanism which leads to the stimulation of mitochondrial energy metabolism following treatment with resveratrol is presently a matter of controversy^67^. AMPK is a key sensor of cellular energy metabolism. Low doses of dietary resveratrol activates AMPK in intestinal mucosa of mice fed with a high fat diet^17^. This activation is extremely rapid, with a detection of activated AMPK only 30 min after resveratrol ingestion that persists only 2 hr before declining^17^. Oral administration of resveratrol also activates AMPK in muscle of healthy obese men^68^. Furthermore, resveratrol enhances brown adipose tissue formation and function by activating AMPK in mice^69^. Altogether, these observations clearly underline the ability of dietary-relevant doses of resveratrol to induce AMPK phosphorylation *in vivo*. The activation of AMPK is a well-documented upstream signaling event known to be triggered by resveratrol^67^ at a broad range of doses, suggesting a difference in cell sensitivity to resveratrol, depending on the biological model. For example, AMPK and the downstream mTOR signaling pathway are activated using concentrations as low as 0.01 µM in Apc 10.1 cells derived from adenocarcinomas of Apc^*Min*^ mice after a treatment for 6 days^17^. In contrast, activation of AMPK is also observed after resveratrol exposure for 24 hr to 50 to 200 µM in human non-small cell lung cancer cells^70^ and in esophageal squamous cell carcinoma^71^. Different molecular mechanisms of AMPK activation have been described upon resveratrol exposure. Activation of the NAD-dependent deacetylase SIRT1 by resveratrol could lead to the deacetylation of LKB1 and ultimately to the activation of AMPK^72^. Resveratrol also could inhibit mitochondrial ATP production and, thus, indirectly activate AMPK by a modification of AMP/ATP ratio^11^. Recently, Park *et al*.^73^ showed that resveratrol inhibits the phosphodiesterase enzyme in myotubes through an increase in the level of cAMP, an activation of the cAMP-dependent guanine nucleotide exchange factor Epac-1 and an increase in intracellular Ca^2+^ which leads to the activation of CamKKB followed by that of AMPK^73^. In low nutrient conditions, the Warburg effect was enhanced through a mechanism that involves ROS/AMPK dependent action of PDK^74^. Here, we demonstrate that exposure of cells to resveratrol leads to a shift from glycolysis to OXPHOS via an activation of PDH associated with a rapid activation of AMPK. We found that the pharmacological inhibition of CamKKB partly abolished the effect of resveratrol on the oxidative capacities of colon cancer cells which suggests that resveratrol stimulates glucose oxidation in a CamKKB-dependent manner. Further studies are needed to decipher the role of the CAMKKs on the resveratrol-induced activation of AMPK.

In conclusion, our study provides evidence that a low dose of resveratrol, close to the amount found in the serum of resveratrol-treated human patients, is able to modify cancer cell metabolism in colon cancer cells, with an alteration of lipidomic profile and a reversion of the Warburg effect. Resveratrol clearly increases the oxidative capacities of cancer cells and decreases the glycolysis. We propose that the PDH complex is a novel target for resveratrol through a Ca^2+^/AMPK signaling pathway.

## Materials and Methods

### Reagents

Resveratrol was from Cayman (Interchim, France). A fresh stock solution (200 mM resveratrol) was prepared extemporaneously in DMSO before each experiment.

### Cell culture

Caco2 were provided by Zweibaum’s laboratory^75^, MCF7 and HCT 116 were purchased from ATCC. Caco2 and HTC116 colon cancer cells were seeded at a density of 7.5 × 10^3^ cells/cm^2^ and cultured in Dulbecco’s modified Eagle’s essential medium (DMEM) containing 25 mM glucose and supplemented with 20% fetal calf serum, 1% penicillin–streptomycin and 1% nonessential amino acids. Cultures were maintained in a 5% CO_2_/95% air atmosphere. Culture conditions of human colonic epithelial cells (HCECs) = 1CT and isogenic clones 1CT-RPA have been previously described elsewhere^23, 25, 26^. Briefly, HCECs are maintained at 37 °C in a 5% carbon dioxide CO2 incubator on Primaria dishes (BD Biosciences). The DMEM/Medium 199 (4/1) is supplemented with human EGF (20 ng/mL), hydrocortisone (1 μg/mL), insulin (10 μg/mL), transferrin (2 μg/mL), sodium selenite (5 nM), L-glutamin (2 mM), 2% fetal bovine serum (Life Technologies, Saint Aubin, France), and gentamicin sulfate (50 μg/mL) (all from Sigma, Saint Quentin Fallavier, France). Twenty-four hours after cell seeding, the medium was removed, cells were rinsed with 1X PBS, and DMEM containing 25 mM glucose supplemented with 5% charcoal-treated fetal calf serum, 1% penicillin–streptomycin and 1% nonessential amino acids was added for 24 hr. Then, cells were treated with 10 µM resveratrol or an equivalent amount of dimethylsulfoxide (DMSO 0.01%, control) in the same medium. For MCF7 breast cancer cells, the culture procedure was similar but the medium was also supplemented with 10^−7^ M insulin and depleted in red phenol.

### Assay for cell growth

Cell proliferation was estimated with a colorimetric immunoassay based on the measurement of the incorporation of BrdU during DNA synthesis (Cell Proliferation ELISA, Roche Diagnostics). Cells were treated with 10 µM resveratrol, and 2 hr before the end of the treatment (*i.e*., at 22, 46 and 70 hr), BrdU labeling agent was added at a final concentration of 10 μM and cells were incubated for additional 2 hr at 37 °C. Labeled cells were then measured by ELISA assay as described by the manufacturer.

### Cell cycle phase distribution

Cells were treated with 10 µM resveratrol for 48 hr and then fixed in ethanol (70%).Following the addition of 100 µg/mL RNAse A and 40 µg/mL propidium iodide, cells were incubated for 20 min at room temperature. The cells were then analyzed using a FACS Calibur flow cytometer (BD Biosciences).

### Assay for cell viability (MTS)

Viable cells were determined by measuring the conversion of the tetrazolium salt MTS [3 (4,5-dimethylthiazol-2-yl)-5-(3-carboxymethoxyphenyl)-2-(4-sulfophenyl)-2H-tetrazolium (Promega) to formazan. Cells were treated with 5 µM, 10 µM, 25 µM or 50 µM resveratrol for 48 hr and then incubated with the CellTiter96 Aqueous Reagent® for 1 hr at 37 ◦C. The absorbance was determined with a spectrophotometer at 490 nm.

### Metabolic studies

#### Incubation of cells in the presence of radioactive precursors

For the measurement of the oxidation of glucose and pyruvate, cells (at subconfluence) were treated for 24 and 48 hr with 10 µM resveratrol. The day of the experiment, 10^6^ cells were isolated and incubated for 90 min at 37 °C in 1 ml of Krebs–Ringer phosphate buffer containing 5 mM [U-^14^C] glucose (0.2 mCi/ml, Perkin Elmer) at an isotopic dilution of 1/1000 or 5 mM [1-^14^C] pyruvate (0.2 mCi/ml, Perkin Elmer) at an isotopic dilution of 1/250. CO_2_ was recovered in methylbenzethonium hydroxide after stopping the reaction with sulfuric acid 6 N. The radioactive CO_2_ was counted by liquid scintillation.

The measurement of ^14^C incorporation into cellular lipids following incubation with ^14^C-glucose was performed as described previously^76^. Briefly, after treatment with 10 µM resveratrol for 48 hr, 2 × 10^6^ cells were isolated and incubated for 90 min at 37 °C in Krebs buffer containing 5 mM [U-^14^C] glucose (0.2 mCi/ml, Perkin Elmer) at an isotopic dilution of 1/1000. Lipids were extracted according to the simplified method of Bligh and Dyer^77^ and the ^14^C from radioactive glucose subsequently incorporated into lipids was estimated by counting the radioactivity associated with the chloroform fraction.

For the measurement of glucose utilization by the pentose phosphate pathway, cells (at subconfluence) were treated for 48 hr with 10 µM resveratrol. The day of the experiment, 3 × 10^6^ cells were isolated and incubated for 90 min at 37 °C in 1 ml of Krebs–Ringer phosphate buffer containing 5 mM [C_1_-^14^C] glucose (0.1 mCi/ml, Perkin Elmer) at an isotopic dilution of 1/1000 or 5 mM [C_6_-^14^C] glucose (0.2 mCi/ml, Perkin Elmer) at an isotopic dilution of 1/100. CO_2_ was recovered in benzethonium hydroxide after stopping the reaction with sulfuric acid 6 N. The radioactive CO_2_ was counted by liquid scintillation.

#### Pyruvate dehydrogenase activity

After a 48-hour treatment of cells with 10 µM resveratrol, PDH activity was assayed by the release of ^14^CO_2_ from ^14^C pyruvate in the cell lysate, according to Clot *et al*.^78^. Briefly, 10^6^ cells were lysed in RIPA buffer (1X PBS, 1% NP40, 0.5% sodium deoxycholate and 0.1% SDS) freshly supplemented with 1 mM NaF, 1X protease inhibitors (Complete, protease inhibitor cocktail, Sigma) and 1X phosphatase inhibitors (Phosphatase inhibitor cocktail II, Sigma). Samples (125 µL) were incubated with a mix (187.5 µL) containing 0.125 µCi [1-^14^C] pyruvate (0.2 mCi/ml, Perkin Elmer), 0.25 mM pyruvic acid, 0.25 mM coA, 16 mM MgCl_2_, 0.4 mM CaCl_2_, 0.5 mM βNAD, 10 mM KH_2_PO_4_, 0.1 mM cocarboxylase, and 100 mU phosphotransacetylase. The tubes were sealed immediately with a rubber cap from which was suspended a plastic cup containing a 25 × 75 mm 1MM Whatman paper, and incubated for 20 minutes at 37 °C. At the end of the incubations 100 µL of 6 N H_2_SO_4_ was added. Hydroxide hyamine (250 µL) was injected with a syringe through the rubber cap onto the paper, which allowed the absorption of CO_2_ and the tubes were shaken gently at room temperature for 1 hr. The filters were then removed and radioactivity was counted by liquid scintillation.

#### Lipidomic studies

Lipidomic analysis was performed according to the previously described method^79^, on control (n = 12) and resveratrol-treated Caco2 cells (10 µM, 48 hr) (n = 12). Briefly, lipids extracts were analyzed by ultra-performance liquid chromatography coupled to high resolution mass spectrometry (UPLC-HRMS) on a Aquity^TM^ UHPLC^TM^ - Synapt^TM^ G2 HDMS^TM^ system (Waters MS Technologies, Manchester, UK). The data obtained were analyzed through an unsupervised principal component analysis (PCA) and supervised partial least squares discriminant analysis (PLS-DA) and orthogonal partial least squares discriminant analysis (OPLS-DA) using SIMCA-P + software version 13.0.3 (Umetrics, Umeå, Sweden). Identification of lipid species was based using mass on charge ratio and retention time obtained in UPLC-MS using the online data bases LIPID MAPS and METLIN and in-house data base, respectively and MS/MS experiments.

#### Analysis of oxygen consumption and extracellular acidification rates

HCEC cells (1CT and 1CT-RPA cells) were seeded in Seahorse XF 24-well microplates (Proteigene, St Marcel, France) at 8 000 cells/well. Twenty four hours later, cells were treated with RES 10 µM for 48 hr. On the day prior to the experiment, XF extracellular flux cartridge was hydrated with XF calibrant overnight. After a 48 hr-treatment, the medium was changed to assay medium (unbuffered DMEM with 10 mM glucose, 2 mM glutamine, 2 mM pyruvate) and kept 1 hr in a non-CO_2_ incubator at 37 °C. Oxygen consumption rate (OCR) and extracellular acidification rate (ECAR) were measured and normalized according to protein content.

### Protein analysis

Cells were treated with 10 µM resveratrol for 48 hr. Proteins were extracted in RIPA lysis buffer (1X PBS, 1% NP40, 0.5% sodium deoxycholate and 0.1% SDS) freshly supplemented with 1 mM NaF, 1X protease inhibitors and 1X phosphatase inhibitors. The cells were extracted on ice for 30 min. The lysates were centrifuged and the supernatants were transferred into new vials. Protein concentration was measured using the bicinchoninic acid method. Twenty micrograms per lane of total proteins were resolved on a NuPAGE 4–12% gel (Fisher Scientific) and transferred to a Hybond ECL membrane (Fisher Scientific). The following primary antibodies were from Abcam: P-PHE1α(S232), P-PDHE1α (S293), P-PDHE1α(S300), PDHE1α, GLUT1, PDK1, PDP1, PDP2, G6PD and β-actin. Antibodies against PKM2, P-PKM2(Y105), LDHA, P-LDHA(Y10), AMPK and P-AMPK(T172) were from Cell Signaling. The primary antibodies were used at a dilution of 1:1000 except for G6PD (dilution 1:500) and PDP1 (dilution 1:300). For AMPK and P-AMPK(T172), the detection was performed using a horseradish peroxidase–linked secondary antibody from Cell Signaling (dilution 1:1000) using the chemiluminescent reagent ECL (Fisher Scientific). Immunoreactive bands were quantified using an ImageQuant LAS4000 (GE Healthcare) and the results analyzed by ImageJ (National Institutes of Health). For the other antibodies, the detection was performed with an IRDye-linked secondary antibody (dilution 1:10.000). The immunolabeled proteins were detected using an Odyssey Infrared Imaging System (LI-COR) and the results were analyzed with the Odyssey Application Software to obtain the integrated intensities. The results were expressed as arbitrary units normalized to the amount of β-actin.

### Statistics

The results are expressed as the means ± SEM. Comparisons between experimental groups were performed by the nonparametric Kruskal–Wallis test or the parametric Fisher test. When a significant difference *p* < 0.05 was found among groups, multiple pairwise comparisons were made following the Kruskal–Wallis or Mann-Whitney method. Statistical tests were performed using the StatView 4.01 Non-FPU (Abacus Concepts) statistical package.

## Electronic supplementary material

Supplementary Information
