# Supplementary material for: Resveratrol reverses the Warburg effect by targeting the pyruvate dehydrogenase complex in colon cancer cells
Source: Sci Rep. 2017 Jul 31;7:6945. doi: 10.1038/s41598-017-07006-0 (PMC5537345; doi:10.1038/s41598-017-07006-0)
Supplement: Supplementary file 1 — Supplementary Information [file 41598_2017_7006_MOESM1_ESM.doc]

**SUPPLEMENTARY INFORMATION**

**Resveratrol reverses the Warburg effect by targeting**

**the pyruvate dehydrogenase complex in colon cancer cells**

Elise Saunier^1,2^, Samantha Antonio^1,2^, Anne Regazetti^3^, Nicolas Auzeil^3^, Olivier Laprévote^3^, Jerry W. Shay^4^, Xavier Coumoul^1,2^, Robert Barouki^1,2^, Chantal Benelli^1,2^, Laurence Huc^5,6^, and Sylvie Bortoli^1,2,*^

**Supplementary Materials and Methods**

#### RNA analysis

Cells were treated with 10 µM resveratrol for 8, 24 and 30 hr. Total RNA was extracted from the Caco2 cells using SV Total RNA Isolation System (Promega). Sample quality was assessed by the 260/280nm absorption ratio (between 1.8 and 2.0) and checked on 1% agarose gel.

For real-time RT-PCR analyses, 500 ng total RNA was first reverse transcribed using the High Capacity cDNA Archive kit (Applied Biosystems). Reverse transcribed RNA were amplified on a thermal cycler (ABI prism 7900 HT, Applied Biosystems) using the SYBR green fluorescence method. The primers used were as follows: RPL13A fwd 5′-CCT GGA GGA GAA GAG GAA AGA GA-3′, rev 5′-GAG GAC CTC TGT GTA TTT GTC AA-3′; human PDK1 fwd 5′-GAT GTG AAT GGG CAG TTA GTC-3′, rev 5′-AAG GAA TAG TGG GTT AGG TGA G-3; human PDP1 fwd 5’-TGG TGT TGG CTA CTG ATG G-3’, rev 5’-ATT GGC TGT TGG TGA TGC-3’; human PDP2 fwd 5’-CAG TTC ACA CCC CCA CAC-3’, rev 5’-CAC CAC CAG CCT TAC CAC-3’. Results were analyzed with the SDS 2.1 real-time detection system software. Quantification of RNA was carried out by comparison of the number of cycles required to reach reference and target threshold values (δ–δCt method).

#### Metabolic studies

*Lactate assay*

Lactate secretion was determined using a colorimetric system as described by the manufacturer (Biovision/Clinisciences). Cells were treated at subconfluence for 48 hr with 10 µM resveratrol. Two hours before the end of the treatment, cells were incubated in Krebs–Ringer phosphate buffer containing 25 mM glucose for 2h and lactate concentrations were measured directly in Krebs–Ringer phosphate buffer after metabolic studies. The corresponding OD570 nm absorption values were plotted against a lactate standard curve to determine the lactate concentrations in samples.

*ATP measurement*

ATP production was estimated by using an ATP Bioluminescence assay kit II (Roche Diagnostics). The day of the experiment, cells were rinsed with 1X PBS. Cells were treated with 10 µM resveratrol for 48 hr. The day of the experiment, cells were rinsed twice with 1X PBS and then 10^6^ cells were lysed. The bioluminescence values were plotted against an ATP standard curve to determine ATP concentrations.

**Supplementary Figure S1 :**

**Supplementary Figure S1: Resveratrol modifies glucose oxidation depending on the dose**

After treatment with 1 to 50 µM resveratrol for 48hr, oxidative capacity was evaluated as following : subconfluent cancer cells were isolated and 10^6^ cells were incubated in Krebs-Ringer phosphate buffer supplemented with 5 mM [U^14^C]-glucose (isotopic dilution 1/1000). ^14^CO_2_ was recovered as described in the Material and Methods section. ). Results are the means ± SEM of at least 3 independent experiments each performed in triplicate (**p<0.01).

**Supplementary Figure S2 :**


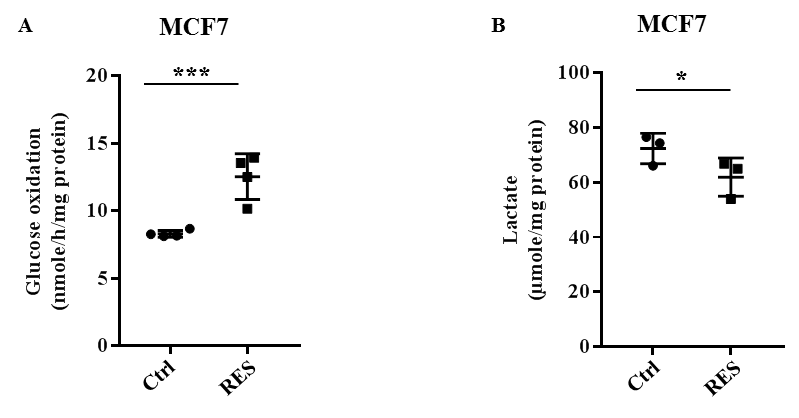


**Supplementary Figure S2: Resveratrol induces a shift toward oxidative metabolism in cancer cells**

A) After treatment with 10 µM resveratrol (RES) for 48hr, oxidative capacity was evaluated as following : subconfluent MCF7 breast cancer cells were isolated and 10^6^ cells were incubated in Krebs-Ringer phosphate buffer supplemented with 5 mM [U^14^C]-glucose (isotopic dilution 1/1000) ^14^CO_2_ was recovered as described in the Materiel and Methods section (n=4). B) The lactate secretion in the medium was measured after an incubation of cells in Krebs-Ringer phosphate buffer supplemented with 5 mM glucose as described in the Materiel and Methods section (n=3). (* p< 0,05, ***p<0.001 vs control).

**Supplementary Figure S3 :**


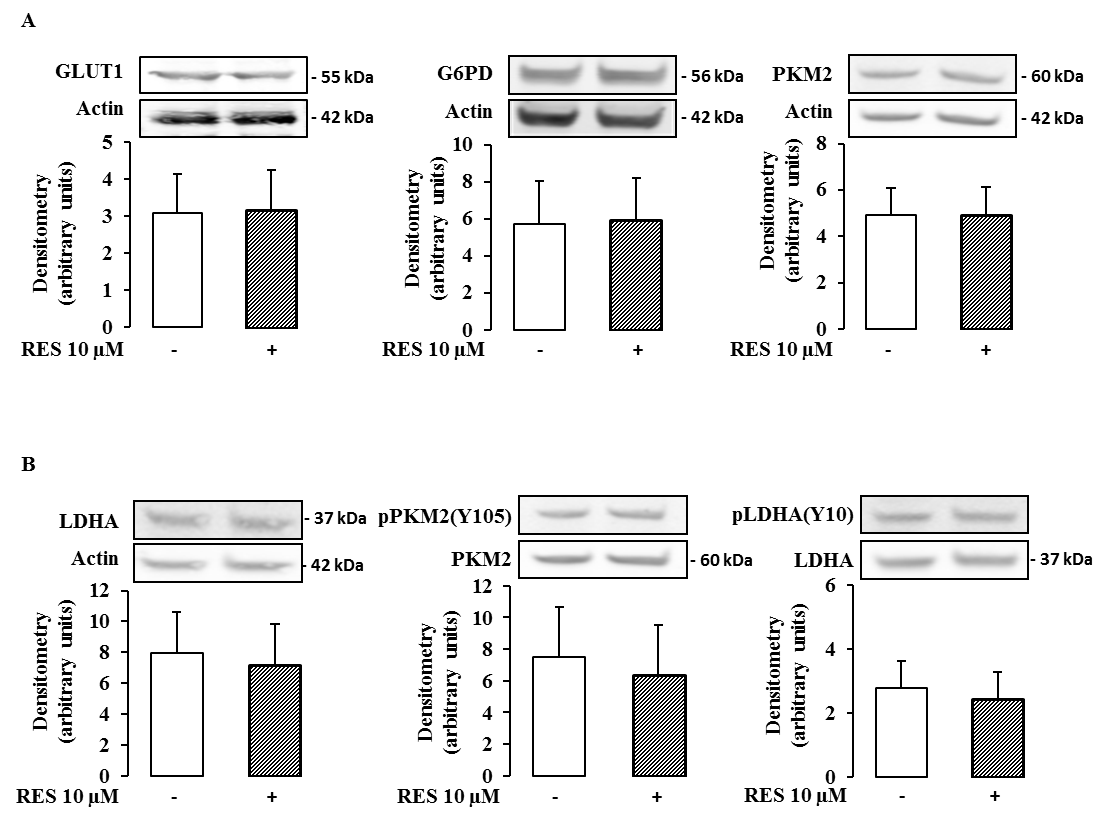


**Supplementary Figure S3: Resveratrol treatment does not modify the expression of some proteins involved in glucose utilization.**

Caco2 cells were cultured at subconfluence in 5% charcoal-treated serum in DMEM 24 hrs before and during treatment with 10 µM resveratrol (RES) for 48hr. Total cell lysates were collected using RIPA buffer supplemented with protease and phosphatase inhibitors and analyzed by Western blot using the indicated antibodies. The results are representative of at least three independent experiments.

**Supplementary Table S1 :**

**Supplementary Table S1 : Identification of lipids showing changes in their relative abundance in Caco2 colon cancer cells after a treatment with resveratrol (10 µM, 48hr).**

**t_R_** is expressed in min, ***m/z*** is given for the most intense adduct. For each lipid, **R** represents the ratio between the level of a lipid in resveratrol-treated cells and that of control cells. p-value were determined by FDR test (5%), *<0.05.
